# Alternatives to Biological Skin in Permeation Studies: Current Trends and Possibilities

**DOI:** 10.3390/pharmaceutics12020152

**Published:** 2020-02-13

**Authors:** Rabin Neupane, Sai H.S. Boddu, Jwala Renukuntla, R. Jayachandra Babu, Amit K. Tiwari

**Affiliations:** 1 Department of Pharmacology and Experimental Therapeutics, College of Pharmacy and Pharmaceutical Sciences, University of Toledo, Toledo, OH 43614, USA; Rabin.Neupane@rockets.utoledo.edu (R.N.); amit.tiwari@utoledo.edu (A.K.T.); 2 Department of Pharmaceutical Sciences, College of Pharmacy and Health Sciences, Ajman University, Ajman 346, UAE; s.boddu@ajman.ac.ae; 3 Department of Pharmaceutical Sciences, School of Pharmacy, High Point University, High Point, NC 27240, USA; 4 Department of Drug Discovery and Development, Auburn University, Auburn, AL 36849, USA; ramapjb@auburn.edu

**Keywords:** transdermal, PAMPA technique, EpiDerm^®^, reconstructed skin models, Strat-M™

## Abstract

The transdermal route of drugs has received increased attention in recent years due to numerous advantages over the oral and injectable routes, such as avoidance of the hepatic metabolism, protection of drugs from the gastrointestinal tract, sustained drug delivery, and good patient compliance. The assessment of ex vivo permeation during the pharmaceutical development process helps in understanding the product quality and performance of a transdermal delivery system. Generally, excised human skin relevant to the application site or animal skin is recommended for ex vivo permeation studies. However, the limited availability of the human skin and ethical issues surrounding the use of animal skin rendered these models less attractive in the permeation study. In the last three decades, enormous efforts have been put into developing artificial membranes and 3D cultured human skin models as surrogates to the human skin. This manuscript provides an insight on the European Medicines Agency (EMA) guidelines for permeation studies and the parameters affected when using Franz diffusion cells in the permeation study. The need and possibilities for skin alternatives, such as artificially cultured human skin models, parallel artificial membrane permeability assays (PAMPA), and artificial membranes for penetration and permeation studies, are comprehensively discussed.

## 1. Introduction

Transdermal delivery has evolved as an effective alternative to the systemic delivery of drugs across the unbroken skin. Some of the major advantages of the transdermal delivery system are: avoidance of first-pass metabolism, possibility of controlled and long-term release, and patient compliance in various medical conditions [1]. However, drug molecules have to penetrate through the stratum corneum, which acts as a formidable barrier to permeation for high molecular weight drugs (>500 Da) and drugs with inadequate solubility in water and oil. Further, the delivery system should not impede the natural barrier properties of the skin. Transdermal drug delivery systems (TDDS) can be classified into three different generations, viz., the first generation comprising of low molecular weight/potent drugs (molecular weight of around 200–500 Da and a log *P_octanol/water_* value between 1 and 3), the second generation comprising of chemical penetration enhancers, and the third generation comprising of physical techniques to overcome the barrier property of the skin [2,3,4,5,6].

**Figure 1:**
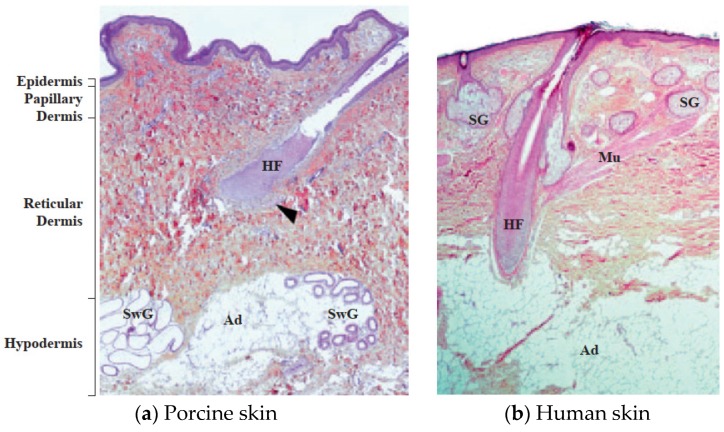
Comparative histological aspect of porcine (**a**) and human (**b**) skin (haematoxylin-eosin-saffron staining). HF: hair follicle, Mu and arrowhead: arrector pili muscle, SwG: Sweat gland, SG: sebaceous gland, and Ad: Adipocytes (hypodermis). Reproduced with permission from Debeer et al. [19].

The suitability of a transdermal system is generally demonstrated using a permeation study. Franz diffusion cells are widely used to determine the drug permeation through the skin. The skin permeation study across the dermatome human skin explants is considered as the gold standard for assessing the delivery of drugs from a transdermal system. However, ethical and economic reasons pose a major problem to the availability and use of human skin. The skin is usually collected for graft applications for use in patients with burns and injuries and stored in frozen conditions using cryo-preservatives such as propanediol or glycerol in normal saline [7,8,9]. Due to long-term storage, the skin lacks viability and enzymatic activity, resulting in variations in the skin permeation [7,10,11,12]. Isolated skin from inbred animals such as porcine; primates; rodents (guinea pig, rat, and mouse); rabbit; and shed snake skin have been routinely considered as alternatives to human skin, as they can be obtained easily, can be excised fresh prior to skin permeation studies with viability and enzymatic activity, and exhibit less variability [13,14]. Porcine skin is generally preferred due to its structural similarity to the human skin in terms of: (1) hair growth density (~20 hairs/cm^2^) and the presence of structures such as Langerhans cells and rete ridges; (2) stratum corneum thickness and contents such as glycosphingolipids and ceramides; (3) stratified, multilayered, keratinizing epithelium (Figure 1); (4) thickness of the viable epidermis (~70 µm); and (5) collagen fiber arrangement in the dermis. Hence, freshly excised porcine skin (dermatome or full thickness), as well as the isolated porcine epidermis, have been routinely used in assessing the skin permeation of transdermal drug delivery systems [15,16,17,18,19].

Nevertheless, obtaining a sufficient supply of excised animal skin could be at times challenging and expensive. Moreover, biological membranes (both human and animal skin models) require a complex preparation process with storage limitations and often result in variability [20]. In an attempt to overcome the limitations of biological membranes, researchers have started using synthetic membranes as a substitute, depending on the purpose, availability, and suitability of the experimental design [21,22]. The thickness of artificial and synthetic membranes is uniform, and they require a simple preparation process with a low storage space. This review aims to summarize recent studies where the skin surrogates were used in the permeation. The pros and cons of using skin surrogates over human skin in permeation studies are summarized from the published literatures. The regulatory requirements for permeation studies based on the European Medicines Agency (EMA) are briefly included in this review. Further, the process parameters of the permeation study that cause a substantial effect on the permeation results are discussed in detail.

## 2. Skin

The skin is the largest organ in covering the body. It acts as a barrier, preventing the penetration of foreign molecules into the body and the loss of water from the body. The skin is made up of three distinct layers: the outermost being the epidermis, followed by the dermis and hypodermis (includes subcutaneous fat). The stratum corneum forms the outermost hydrophobic layer of the epidermis, with 10–30 µm thickness, and acts as a prominent barrier for skin permeation. The barrier property of the stratum corneum is due to its extreme lipid component and corneocytes, which are filled with keratin filaments and filaggrin. The corneocytes are embedded in the dense structure of the multilamellar lipid, comprised of lipid-like sterols, phospholipids, and glycosphingolipids (ceramides). The corneocytes embedded in the lipid matrix are well-described as a brick and mortar model [23,24,25]. The stratum corneum of the excised human skin loses its integrity/viability if stored for a long time. Ideally, an excised human skin is viable for a period of eight days after excision, if stored at 4 °C [26].

**Figure 2:**
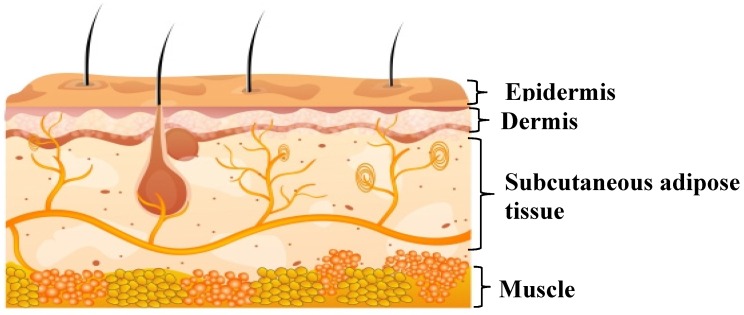
Anatomy of the skin. Credit: Image courtesy of digital art at FreeDigitalPhotos.net.

Beneath the stratum corneum, viable layers like the stratum granulosum, stratum spinosum, and stratum basal are present. The stratum basal is the layer which is actively proliferating via mitosis. The cell division in the basal layer eventually moves outwards towards the stratum corneum. During this period, the cells undergo morphological and histochemical changes (keratinization) to form the stratum corneum. Therefore, the loss of cells from the stratum corneum is compensated. The dermis is the skin layer with extensive blood circulation [27]. The capillaries in the dermal layer provide the sink condition for the molecules permeating through the transdermal route. Further, blood circulation is the key to maintaining skin temperature, for the supply of oxygen and nutrients, and to remove the toxins out from the skin. Below the epidermis, there are layers of hypodermis comprising the subcutaneous fat tissue (Figure 2). The hypodermis possesses the blood vessels, nerves, and regulates the temperature. This layer provides mechanical support to the epidermal and dermal layers.

The routes of permeation of the drug across the skin in the vascular region of the dermis are broadly classified as: (1) the transcellular route (diffusion across corneocytes); (2) the intercellular route (diffusion across the lipid matrix); and (3) the shunt pathway or appendageal route (diffusion into the sweat gland, hair follicles, and sebaceous gland). In the transcellular route, the drug encounters the low lipid regions in the cytoplasm of the corneocytes, whereas the intercellular lipid matrix comprises of dense lipids like ceramides and fatty acids. Therefore, this pathway possesses both hydrophilic and lipophilic regions, making this route very resistant to drug permeation. In the intercellular route, which involves a tortuous pathway along with the lipid lamellae, the lipid lamellae possess a polar head, which favors permeation of hydrophilic molecules, and lipophilic molecules make use of the lipid tail [28]. In the shunt pathway, permeant molecules and nanoparticles travel through the sweat ducts and hair follicles. The surface area occupied by the appendages is only about 0.1% of the skin continuum; hence, this route is considered as a minor route for transdermal permeation. The nanoparticulate carries can seep through appendages and serve as reservoirs for the local delivery of drugs in skin disorders [29].

## 3. Permeation Study

### 3.1. Summary of European Medicines Agency Guideline (EMA) for Permeation Study

According to the EMA, in vitro permeation studies are meant to evaluate the permeation of drugs across the skin layers. Moreover, the EMA does not necessarily expect to establish a correlation of permeation results to that of in vivo permeation. For the in vitro permeation study, use of the human skin or skin from species like a pig, rodent, guinea pig, or artificial/synthetic membrane is acceptable, until the model is justified. For the purpose of comparison of a generic product, the same membrane or skin should be used. The EMA recommends typically six or more replicates from at least two or more donors in the study to minimize the skin variability. When biological skin is used, then the species detail and the body part from where the skin is obtained should be specified. The storage condition and thickness of the skin used should be explained and justified. The skin integrity should be tested and justified in the beginning of the experiment. Methods such as transepithelial electrical resistance (TEER), transepidermal water loss (TEWL), and permeation of tritiated water should be used to verify the skin integrity. A surface area ranging from 0.5 to 2 cm^2^ in the Franz diffusion cell is accepted. The Franz cells used in permeation studies are of two types: commercially manufactured Franz cells (expensive and provide the least variability) and hand-blown Franz cells (cheap and variability depends on the technician’s skill). The receptor media in the permeation study should be an aqueous buffer for water-soluble drugs, while for poorly water-soluble drugs, hydroalcoholic media or aqueous buffers with solubility enhancers such as protein solutions are used. The receptor solution should not affect the skin or membrane integrity used in the study. Therefore, the use of surfactant in the receptor media is limited by its potential to affect the skin integrity. It is essential to maintain the sink condition in the receptor chamber such that the drug concentration should not exceed 10–30% of the maximum solubility of the receptor solution [30,31]. The number of sampling points should be five or more, which are suitably timed to represent the permeation. The samples at the end of the permeation study can be analyzed using a validated analytical method, such as high-performance liquid chromatography (HPLC) or liquid chromatography-mass spectrometry (LC-MS) [30].

### 3.2. Experimental Parameters Affecting the Permeation Study

During the permeation study, it is essential to maintain the sink condition in the receptor compartment of the Franz diffusion cell. An ideal sink condition would have a zero-drug concentration in the receptor medium. However, it is not practically possible to achieve a perfect sink condition with static diffusion cells. The experimental setup conditions of Franz cells have a great influence on the results while carrying in vitro permeation studies [32]. The variation in permeability data is influenced by experimental parameters like temperature, sampling frequency, stirring condition, and membrane chemistry [33].

The passive diffusion of drug substances is affected by the membrane temperature and diffusion chamber. An in vitro permeation study is carried out at a constant temperature of 32 ± 1 °C, with humidity between 30% and 70%. The temperature alters the fluidity of stratum corneum lipids, resulting in a transition from the gel to a liquid crystalline state [34,35,36]. Akomeah et al., demonstrated that the flux value of compounds such as methyl paraben, caffeine, and butyl paraben was increased by two-fold with a 7–8 °C rise in the temperature of the receptor medium [37]. A temperature-controlled water bath is used to maintain the temperature of the Franz cells during permeation studies. The solubility and diffusivity of the drug is highly influenced by the temperature. Moreover, the percutaneous flux almost gets doubled with a rise in temperature by ~10 °C [38]. Therefore, it is important to consider the temperature fluctuation incurred during the study.

The topical application of drug substances should mimic the specific condition (local delivery versus systemic absorption) on a controlled surface area, and excess drug substances must be cleansed with a relevant agent for analysis. The removal procedure of the test preparation should be justified based on the expected use condition. An adequate absorption profile requires a 24-h sampling during which the skin barrier function should remain intact. When skin is used in permeation studies, the barrier integrity of the skin is an important parameter to be determined prior to the study. According to regulatory requirements like the Organization for Economic Cooperation and Development (OECD) 428, the test for skin integrity can be performed using different instruments like TEER, tritiated water-flux (TWF), and TEWL [39]. TEER is the prevailing method for the testing of skin integrity, as it is simple, quick, safe, and cost-effective. While using TEER, the current used, frequency of the current, the equipment in measuring resistance, and even the diffusion cells affecting the skin surface could potentially affect the output of the TEER measurement [40]. Davies et al., have demonstrated the robustness of the TEER method in comparison with the TWF method. In this study, they proposed the TEER values for intact skin in six different species. The TEER value should be ≥10 kΩ for human, 5 kΩ for mouse and guinea pig, 4 kΩ for pig, 3 kΩ for rat, and 0.8 kΩ for rabbit [41]. The TEWL is also a popular method for skin integrity. TEWL is noninvasive in nature and can be used in both in vivo and in vitro assessments of skin integrity. In the TEWL method, the water vapor flux above the stratum corneum is measured, which is an indicator of water diffusion through the stratum corneum and its barrier property. Importantly, the water vapor flux above the stratum corneum is influenced by the microenvironment adjacent to the skin. Therefore, TEWL requires time for stabilization in a controlled temperature and humidity environment around the probe [42]. An impaired barrier condition of the skin could be due to disease conditions (psoriasis, skin irritation, or sensitization) or sunburns in which the TEWL can be elevated by several folds [43,44]. In a stable ambient condition, the human skin TEWL oscillates around 4–10 g/m^2^/h, depending on the skin area, but it may increase even up to a 30-times higher value when the epidermis is damaged [45]. Davies et al., have reported a method for damaging the barrier properties of the skin by tape stripping prior to the permeation study. With the increasing number of tape strippings (5–20 tape strips), there was a gradual reduction in the TEER value, whereas increment in TEWL and TWF values indicate the loss of barrier integrity of the skin. A clinically compromised skin has a TEWL value increased by 3–4-fold, which can be achieved after five tape strips on the skin. Therefore, for an in vitro study, the skin after five tape strips can mimic skin with compromised barrier function [46,47]. Generally, two dosing regimens are used in in vitro permeation studies, namely, finite dosing and infinite dosing. In infinite dosing, there is no change in the permeant concentration within the formulation throughout the experiment, while in finite dosing, permeant concentrations in the formulation changes during the experiment. In finite dosing, ≤10 µL cm^−2^ of a liquid (or) 1–5 mg cm^−2^ of a solid formulation is applied, and in infinite dosing, amounts of >100 µL cm^−2^ or >10 mg cm^−2^ are used [48]. Fick’s laws of diffusion describe the transdermal permeation experiment. The flux and apparent permeability are calculated by using the following Equation:

Permeability (P_app_) = Flux/C_d_

Flux (J) was calculated by dividing the slope obtained by plotting the cumulative amount of drug permeated (M) through the skin vs. time (t) with the cross-sectional area of the membrane (A) exposed to the drug. C_d_ was the initial drug concentration in the donor chamber [49].

Sampling time points should be placed at a suitable distance from each other to obtain an absorption profile of the drug substance. For test substances that penetrate slowly, longer exposure times may be required. The receptor fluid should be continuously agitated with the help of a magnetic stirrer at an optimum stirring speed to facilitate proper mixing. Moreover, the receptor fluid should remain in contact with the bottom side of the membrane with no air bubbles. Insufficient stirring (e.g., fast stirring at the base, but negligible stirring in the upper region) might be a reason for the deviation in sink conditions. The conditions like vortex formation due to excessive stirring causes undesirable effects like disruption of the static receptor fluid layer affecting the diffusion according to the Fick’s law [50,51]. Proper care should be taken to avoid the bubble formation underneath the membrane surface. Sampling can also generate air bubbles below the membrane, which might cause a transient temperature drop. Bubbles should be removed from the side arm by tilting the Franz cell.

Similar permeation profiles and flux values can be obtained on different days by controlling the parameters like stirring, temperature, cell type, and operator. For example, Akomeah et al., have demonstrated a similar epidermal flux (*p* > 0.05) on different days for three different compounds with varying physiochemical properties when all parameters were kept the same [52]. For six replicates, the use of validated equipment and methodology in the permeation study reduced the coefficient of variation from 25.7% to 5.3%. This study demonstrated the potential of validation to avoid the variation in intra-and inter-lab replicates in the permeation study with a smart membrane. An inter-laboratory permeation study involving 10 laboratories using the porcine skin estimated that 13% of error in the result is attributed to the operator of the Franz cell experiment. Fifty-Five percent error was observed due to the skin and 45% from the experimental conditions [53]. Therefore, for reliable results in the in vitro percutaneous study, experimental parameters and skin should be given major consideration.

### 3.3. Significance of In Vitro Permeation Study Using Excised Human Skin

In the early stage of transdermal formulation development, an in vitro model with human skin is considered as an important benchmark for the prediction of percutaneous absorption. An in vitro study using an excised human skin model gives an idea of the potential steady state concentration following the percutaneous absorption of drugs. The extent to which in vitro permeation truly mimics the human body with glandular secretion, metabolic activity, and circulation still questions the authenticity of the in vitro permeation study. Historically, there are studies that have shown a strong in vitro-in vivo correlation for the transdermal permeation. For example, excised human skin was used as a surrogate for in vivo studies of compounds like caffeine and benzene. The commercially successful products like Transderm-Scop^®^, Androderm^®^, and Alora^®^ were developed eventually with a strong in vitro-in vivo correlations [54]. Therefore, the role of in vitro permeations using an excised human skin in the determination of bioavailability is apparent. Despite several examples of in vitro-in vivo correlations, the use of an in vitro model as a surrogate for clinical trials is still questionable. Likewise, for the generic product development, establishment of bioequivalence requires clinical data. A clinical study requires a lot of investment and time compared to in vitro studies. So far, a vasoconstrictor (VC) assay for topical glucocorticoids is the only surrogate method of clinical trial approved by the US Food and Drug Administration (FDA). Other methods like in vitro dermatopharmacokinetic methods in which the permeation is determined by using the excised human skin are considered as potential surrogates by the FDA [55]. However, they were withdrawn later due to inconsistency observed in inter-laboratory validations. Recent studies have shown the usefulness of an in vitro permeation study using the human excised skin as a potential tool for the establishment of bioequivalence. Franz et al. [54] demonstrated a strong in vitro and in vivo correlation of six different generic products. The ratio of the percutaneous absorption of six generic drugs (test) to the marketed product (reference) was approximately one, which indicated the bioequivalency of the products. The clinical trial data were in coherence with the in vitro permeation data. Further, the author claimed that the in vitro model could discern the difference in the concentration of APIs between the formulations based on their observation of a study using tretinoin (a poorly soluble drug). There is a convention that the permeation result in such poorly soluble compounds in an in vitro model is erroneous, if the full thickness of skin is used with isotonic saline in the receptor medium [56]. In this study, the authors have demonstrated the use of dermatome skin and 0.5% Volpo-20 in the receptor medium to overcome such challenges. Neither the solubility nor the skin thickness affected the study results. Still, the suitability of the use of Volpo-20 in the receptor medium is to be established. In conclusion, the in vitro test using human skin can potentially replace the expensive clinical trials required for bioequivalence following the approval by the regulatory authorities.

### 3.4. Effect of Drug Properties on the Permeation Mechanism Through the Skin

The drug molecule permeation through the skin is an intriguing phenomenon. Four possible routes of permeation have been suggested: i.e., free volume diffusion through lipid bilayers, diffusion through the pores, lateral diffusion along lipid bilayers, and diffusion through shunts. The physiochemical characteristics of a molecule are crucial to define the tendency of permeation across the skin layers. Low-Molecular weight hydrophobic solutes with molecular weights less than 400 Da undergo permeation following free volume diffusion through the lipid bilayers by hopping between the free volume pockets. The density fluctuation in the lipid chain results in free volume pockets of radius 4 Å with lifetime ~1.6 μsec. Large hydrophobic molecules partition preferentially in the lipid bilayers. Further large-sized hydrophobic molecules have a low diffusion coefficient because of their large size. The time required for large hydrophobic drugs to jump through the lipid pocket exceeds the lifetime of pocket formation. Therefore, the free volume diffusion theory is not applicable. For molecules with molecular weights greater than 400 Da, the permeation majorly occurs via lateral diffusion of lipid molecules [57]. Marqusee and Dill have shown the steric expulsion of drug molecules from a highly ordered lipid chain structure in the interfaces. Therefore, the partition coefficient of drug molecules is minimal at highly organized bilayers but tends to be high at the center of the bilayers [58]. Further simulations were used in the permeability study of hydrophobic molecules across the local structure of the lipid bilayer. These studies also revealed that the permeation of such molecules is affected by the steric expulsion of the solute in the interface of the lipid bilayer [57,59,60].

On the other hand, hydrophilic molecules undergo permeation preferentially through the shunt pathway or via diffusion through pores of the stratum corneum. The pores on the skin surface originate from defects or imperfections. The phenomenon like hydration causes swelling and fluidization of the stratum corneum, thereby increasing defects within the stratum corneum. The stratum corneum comprises of components like ceramides, cholesterol, and fatty acids. Due to the presence of different components, the packaging defect leads to the pore formation. Unlike the hydrophobic molecules, a hydrophilic molecule passes through pores without any interaction with the lipid bilayers, while a hydrophobic molecule requires a continuous lipid bilayer for permeation. The pores through which the hydrophilic molecules preferentially undergo permeation occupies a certain area in the skin. Such small areas of defects in the skin will not create discontinuity of the lipid bilayer. The porosity, tortuosity, and pore size distribution are three parameters affecting permeation through pores. Porosity is an innate characteristic of the skin, whereas the tortuosity depends on the size of the drug molecules. The smaller the size of the drug molecule, the higher is its ability to access into a large fraction of pores in the skin. Therefore, the tortuosity for the small molecules will tend to be larger than for the drug molecules with larger radii [57]. A drug molecule might follow more than one pathway during permeation. Therefore, the skin permeability of a drug molecule, which is hydrophilic or lipophilic, can be mathematically described using the following Potts and Guy’s Equation [61]:

log *P* (cm/sec) = α log *K* − β MW + δ

where *P* is the in vitro permeability coefficient of drug molecules in an aqueous solution through the human skin; *K* represents the octanol-water partition coefficient; MW represents the molecular weight; and α, β, and δ are constants.

Caffeine is a model hydrophilic compound that has been widely used in transdermal permeation studies. The permeation of polar molecules like caffeine is affected by the inhomogeneity of skin features like the lipid content in the stratum corneum, pores or defects in the stratum corneum, distribution of follicles, and glands of the skin (sweat and sebaceous gland) [57,62,63]. The intracellular lipid in the skin offers a binding site for polar molecules, which facilitates the permeation. For polar molecules that tend to show saturable binding to the polar heads of skin lipids, the permeation will be sensitive to the variation of lipid content across the skin used in the donor compartment. Therefore, inhomogeneity in the skin will result in a higher standard deviation of permeation data [64]. Further, Banning et al., have demonstrated the affinity of polar compounds like doxycycline to the skin components like keratin or the cornified envelope as a reason behind the variation in permeation results [65]. Hayashi et al., studied the effect of donor compartment pH on the permeation of weakly acidic or basic drug in hairless rat skin. Indomethacin was used as a model drug. Indomethacin has a pKa of 4.5 with a high solubility at basic pH. An exponential increase in the steady state permeation rate was observed with increasing pH for indomethacin. This indicates that the ionized fraction of the drug is permeable through the skin. When the permeation is carried out with the donor vehicle in an acidic pH range for a weakly acidic drug, the effect of ionized species in total drug permeability is low. Moreover, if the donor compartment contains an alkaline vehicle and acidic drug, there will be a larger fraction of the ionized drug, and the total permeation of the drug will be affected by the ionized fraction of the drug. The permeability coefficients of ionized and unionized species of indomethacin at various pHs were 1.50 × 10^−7^ and 2.79 × 10^−5^ cm/s, respectively. The contribution of ionized or unionized species in the total permeation should be defined, while the chemical or physical techniques are adopted to enhance the permeation of the formulation [66].

### 3.5. Need of Human Skin Alternatives

Excised human skin is generally used in in vitro permeation studies of topical and transdermal formulations in the USA and the EU. However, its use is limited in Japan. The use of human skin for in vitro experiments can be expensive and pose ethical issues. The availability of a skin source might be challenging. The use of animal tissues instead of human skin is another possibility. Although animal skin costs are far lower than those for human skin, there is variation in animal skin based on the age, sex, and race between or within the same animal skin which could potentially affect the permeability [67]. These issues have led scientists to develop many forms of artificial (synthetic) membranes and culture living skin equivalents to overcome these problems. Synthetic membranes avoid the anatomical variations incurred by the skins in permeation studies. Thus, synthetic membranes exhibit superior in vitro permeation data with less variability. Using the artificial membranes, inferences about partition and the diffusion phenomenon could be made. Nevertheless, the lipid perturbation effect that is exhibited by biological membranes is absent in an artificial membrane [68]. There are restrictions imposed on animal use in the development of cosmeceuticals in the EU by the cosmetic directive 76/768/EEC [69]. The FDA has suggested the use of different polymeric membranes in permeation studies, like polysulfone, polyethersulfone, cellulose, and polydimethylsiloxane [70]. An ideal synthetic membrane for in vitro performance testing should be inert, not occlude the drug penetration, and provide good permeability [71]. Nevertheless, the use of synthetic membranes instead of skin might not reflect the actual drug permeation but just the drug release. For example, Borge et al., used a cellulose acetate membrane in a Franz cell to study the release of dapsone. The release of the drug after 24 h of the study was higher than its permeation through the porcine ear epidermis for dapsone using Franz cells [72]. Moreover, the use of artificial membranes cannot provide an insight into the interaction of the formulation components with skin. Models equivalent to the living skin were developed by culturing human skin components such as keratinocytes and fibroblasts. Such models possess live cells that are mitotically and metabolically active. Currently, there are various marketed skin models, such as EpiDerm™, EpiSkin™, and Labskin™. However, these models have demonstrated higher permeation of drug molecules, as compared to human skin [73]. These models are mostly employed for irritation and toxicity testing. More recent applications of these models in topical and transdermal product testing are discussed in the following section.

## 4. Skin Surrogates in Permeation Studies

Skin surrogates used to determine the permeability of drugs are artificially cultured human skin models, parallel artificial membrane permeability assays (PAMPAs), and artificial membranes based on simple polymeric or lipid models. In these membranes, the in vitro permeation study fails to incorporate components like metabolism, distribution, and excretion that occurs in human in vivo conditions. Still, these are advantageous in terms of membrane homogeneity and providing consistent/reproducible permeation data for the test compounds. These systems are useful for the screening of many test compounds.

### 4.1. Artificial Cultured Human Skin Model

The artificial skin model has been under development for three decades. Initial surrogate models of skin were designed for cutaneous irritancy studies, wherein normal human keratinocytes (NHKs) were grown on de-epidermized dermis [74]. This model was further refined into the reconstructed human epidermis by growing NHKs on supporting membranes [75]. Skin models are broadly classified into two groups: the reconstructed human epidermis (RHE) model and the full human skin model (living skin equivalents (LSEs)). The epidermal models are developed by proliferation of normal human keratinocytes into the multilayered epidermis under suitable conditions. On the other hand, the full-thickness model comprises both epidermal and dermal layers. Commercially available RHEs include: the EpiSkin™ model (L’Oréal, Lyon, France); EpiDerm™ model (MatTek Corporation, Ashland, MA, USA); reconstructed human epidermis (SkinEthic™, Lyon, France); EpiCS^®^ model (CellSystems, Troisdorf, Germany); open source reconstructed epidermis model (Phenion, Düsseldorf, Germany); Straticell model (Straticell, Les Isnes, Belgium); and Labcyte model (Gamagori, Japan). Full-Thickness models such as a StrataTest^®^ model (Stratatech, Madison, WI, USA); Phenion Full-Thickness Skin Model (Phenion, Düsseldorf, Germany); GraftSkin^®^ (Apligraf; Organogenesis, MI, USA); EpiDermFT^®^ (MatTek Corporation, Ashland, MA, USA); and Vitrolife-Skin™ model (Kyoto, Japan) are also available commercially [76]. These models are mostly used in areas such as replacement tissue in burns and bruises, phototoxicity, corrosivity, skin irritancy testing of chemicals, and transdermal permeation studies. In this section, we will limit our discussion to the use of 3D in vitro reconstructed human skin models in drug permeation studies.

Schmook et al., studied skin permeations of salicylic acid, hydrocortisone, clotrimazole, and terbinafine through ex vivo human (dermatome), porcine, and rat skin; GraftSkin^®^ LSE; and SkinEthic™ RHE. GraftSkin^®^ LSE acted as a barrier to salicylic acid; however, more hydrophobic compounds like clotrimazole (e.g., ~900-fold higher flux and 50-fold higher skin concentrations as compared to human skin) easily permeated, even more than rat skin. SkinEthic™ RHE showed similar concentrations of salicylic acid-like human skin with ~seven-fold higher flux. The flux and skin accumulation values of drugs in the following order: human ≤ porcine < rat < GraftSkin^®^ << SkinEthic™ [77]. Later, Dreher et al., evaluated the cutaneous bioavailability of cosmetic preparations containing caffeine or alpha-tocopherol using human skin models (EpiDerm™ and EpiSkin^®^) and compared them with human skin ex vivo at finite doses. The behavior of alcohol-containing vehicles appeared differently in EpiDerm™ and EpiSkin^®^ models compared to the human skin. This was attributed to the less-pronounced barrier properties and increased hydration of the outermost stratum corneum layers of the human skin models [78]. Schreiber et al., reported a similar trend for permeation coefficients of caffeine and testosterone: human < pig < EpiDerm™ << SkinEthic™ [79]. Similar studies were conducted by Schäfer-Korting et al., to study the permeation of caffeine and testosterone across RHE models (EpiDerm™, EpiSkin^®^, and SkinEthic™); human epidermis; and animal skin. The permeation coefficients of testosterone were in the following order: human epidermis, bovine udder skin, pig skin < EpiDerm™, EpiSkin^®^ < SkinEthic™, while, for caffeine: bovine udder skin, EpiDerm™, pig skin, human epidermis < SkinEthic™, EpiSkin^®^ [80]. Further, the same group validated the RHE models (EpiDerm^TM^, EpiSkin^®^, and SkinEthic^®^) using nine different substances (different physicochemical properties) in ten different laboratories using both finite and infinite dose models. While the permeation of drugs through the RHE models exceeded through the human epidermis and pig skin, the order of permeation ranking of the drugs was found to be similar through all membranes. The reproducibility of the permeation parameters in the RHE was in no way better compared to the excised skin. This study concluded that EpiSkin™, EpiDerm™, and SkinEthic™ could be considered as alternatives to human skin and pig skin for in vitro permeation and penetration studies when drugs are applied as aqueous solutions [81]. The Phenion FT model also showed weak barrier properties against benzoic acid and caffeine, whereas it efficiently retarded the permeation of lipophilic compounds such as nicotine and testosterone [82].

From the above-mentioned studies, it is evident that the RHE models, particularly SkinEthic™, were significantly more permeable than biological skins, mainly the human and pig skins. Currently, there are no reliable and established reconstructed skin models (RSM) with barrier functions that matches the human skin. Although the RSM possesses cells like the native skin, the structures like blood vessels, hair follicles, glands, and the lipid organization around the cells are missing. Tfayli et al., used a chromatographic method to elucidate the lipid composition in the stratum corneum of the RSM. Raman spectroscopy was used to evaluate the conformational order, lateral packing, and distribution of the lipids in EpiDerm™. The lipid composition of the stratum corneum in RSM was like that of the human skin stratum corneum, and the only difference observed was in the composition of the ceramides. Unlike the human skin, the Raman spectroscopy of the RSM surface showed a continuous keratin layer on the surface, whereas the cholesterol was present largely as droplets and not evenly mixed with the ceramides and fatty acids. Therefore, the poor barrier property of the RSM is due to the poorly mixed and nonhomogeneously distributed lipid surface, preventing the formation of a continuous lipid barrier [83]. Thakoersing et al., investigated the lipid composition and organization in the stratum corneum of human skin equivalents (HSEs) using the fibroblast-derived matrix model (FDM) and Leiden epidermal model (LEM) and compared these models with the full-thickness collagen model (FTM) and the human skin. The FTM had a higher flux of benzocaine compared to the FDM and LEM, indicating the better barrier property of the FDM and LEM. Nevertheless, the barrier functions of the FDM and LEM were poor compared to human skin. Lipid organization of the stratum corneum can be explained based on the lamellar organization and lateral packing of lipids within the stratum corneum. The lamellar organization of the stratum corneum lipids is basically categorized as a long periodicity phase (LPP) and short periodicity phase (SPP). The HSEs used in the study possessed the LPPs but SPPs were absent. The barrier property of the stratum corneum is a function of LPPs rather than SPPs. The number of LPPs between cells in the stratum corneum of HSEs are higher than that of the stratum corneum of human skin, which would most probably increase the intercellular channels promoting the permeation in HSE. Further, the HSEs of the stratum corneum have a hexagonal packing of lipids rather than orthorhombic packing observed in the human stratum corneum. Enough experimental results were obtained to explain the higher permeability of HSEs compared to human skin. Such difference in the permeability between HSEs and natural human skin is yet to be understood [84]. The literature evidence clearly establishes the popularity and potential of in vitro 3D models containing both normal human keratinocytes and fibroblasts to closely mimic the physiological functions of the human skin. However, the main application of these models, so far, are limited to the assessment of skin irritation due to chemicals/drugs, creams and cosmetics, wound healing, skin ageing, and disease pathology [85,86]. The use of in vitro 3D models in permeation studies is limited due to the differences in lipid composition and organization in comparison to native skin. Improvement in the barrier properties of in vitro 3D models could be accomplished through the regulation of the ceramide subclass composition, decrease in the level of monounsaturated fatty acids/unsaturated ceramides, and increase in the chain length of ceramides/fatty acids [87]. More efforts are needed to develop a well-defined physiological matrix and microenvironment by fine-tuning cell culture media/conditions and including additional cell/tissue components.

### 4.2. Skin Parallel Artificial Membrane Permeability Assay (Skin-PAMPA)

**Figure 3:**
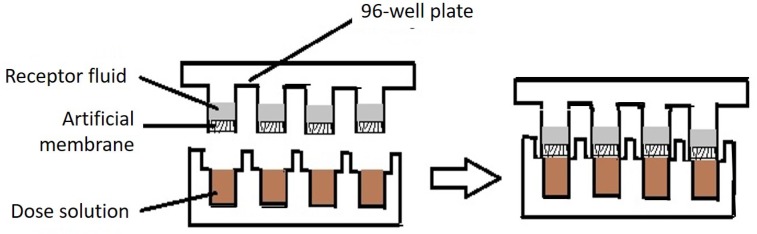
Typical parallel artificial membrane permeation assembly constituting the membrane between the donor and receptor fluid.

In PAMPAs, an artificial liquid membrane separates the donor and receptor compartments in 96-well plates. The receptor compartment consists of a buffer solution, and the donor compartment contains the drug for which permeability is to be determined. A typical PAMPA assembly is shown in Figure 3. The PAMPA was initially designed to estimate passive gastrointestinal absorption and blood-brain barrier permeability. A skin-PAMPA model was developed by adding synthetic certramides, cholesterol, and stearic acid [88]. In comparison to the diffusion cells, the PAMPA is preferred owing to its short time period required for the high-throughput screening of compounds for obtaining a permeation profile. Ottaviani et al., used isopropyl myristate (IPM), silicone oil, and corresponding mixtures as the liquid barrier in the PAMPA experiment to study the permeation profile of 19 compounds with different physiochemical properties. The permeability coefficient obtained from the PAMPA using liquid silicone membranes for eight different compounds was compared with the results obtained by Geinoz et al. [89]. The PAMPA results showed good correlation with the permeability coefficient measured using diffusion cells with a silicone membrane. This indicates that the PAMPA could be a possible alternative to diffusion cells in permeation studies. The liquid silicone membrane allows sufficient permeation of hydrophobic compounds but a poor permeation to polar compounds. Liquid silicone in the PAMPA could be used for screening compounds with diverse permeation profiles. IPM showed poor correlation in permeation coefficient values in comparison to the skin. Therefore, it was concluded that IPM alone could not mimic the skin barrier property in the PAMPA model. Further the mixture of 30% IPM and 70% silicone showed the best liquid artificial membrane mimicking the skin in the PAMPA model. This composition could discriminate the permeability coefficient as human skin permeability, and the correlation between these two parameters was good. The use of IPM in the mixture mimicked the nature of the stratum corneum to accept rather than donate the H-bond [90]. Sinko et al., aimed to develop a PAMPA model with the components of natural skin. The cholesterol, free fatty acids, and the ceramides analogues (namely synthetic certramides, long-chain tartaric acid diamides) were used in the preparation of the artificial membrane. The ceramides are expensive natural extracts, so the analogues of ceramides were synthesized and tested for the permeation. Among different ceramide analogues, ceramides analogue C8–C18 showed good correlation between the permeability of the compounds compared to human skin permeability. This study reported a strong correlation of the PAMPA (containing free fatty acids, cholesterol, and certramides in ratios close to 1) permeability data for 22 compounds with the results obtained from the human skin. Nevertheless, the most abundant ceramides in the human skin are of chain length C24–C26. The skin-PAMPA membrane was shown to be sensitive to the use of penetration enhancers like PEG 400 that is used in the formulation [91]. Luo et al., have demonstrated the superiority of the PAMPA over porcine skin and silicone membranes to discriminate different types of TDD formulations, i.e., gel and solution. This study observed a significant difference in permeation profiles of ibuprofen in the PAMPA from a simple solution and commercial ibuprofen formulations (Ibugel™ and Ibuleve™). Further, this study unraveled an appropriate finite dose in the PAMPA mode, which is 1 µL for ibuprofen. However, the finite dose for other active pharmaceutical ingredients might vary. The permeation profiles from the silicone membrane and the PAMPA were similar, whereas permeation from the human skin was found much lower compared to the PAMPA [92].

Vizserálek et al., investigated the marketed patches of fentanyl, ketoprofen, rivastigmine, and nicotine using Franz cells and the PAMPA. The delivery rate of the drug as per the manufacturer was compared to the results obtained from the PAMPA and Franz cells. The edge effect was prominent in all cases, resulting in a higher permeability profile in both Franz cells and the PAMPA compared to the declared permeability by the producer. The virtual assay surface is increased by the lateral diffusion of API within the adhesive layer of the patch, which is termed as the edge effect. One of the ways to avoid the edge effect is to cut the patch size to avoid overestimation. The standard deviation in the PAMPA was found to be higher than in Franz cells for the local therapeutic patch Keplat^®^. There are two possible reasons for a high standard deviation in the PAMPA. First, the patch has a large surface area, i.e., 70 cm^2^, which covers 6–80 single-well plates, and the permeated amount was calculated based on the average permeation from these well plates. Second, the API within a patch could be nonhomogenously distributed, which does not affect the therapeutic efficacy but can cause a high standard deviation in a PAMPA model. The permeation profile of the patch containing rivastigmine and nicotine (Nicotinell^®^) was found to be identical in both Franz cells and the PAMPA. Further, the ratio of the manufacturer’s labeled in vivo flux value (J_in vivo_) to the maximum flux obtained from the PAMPA (J_max_) study was used in the classification of different types of patches. The first group had the J_max_ equal to J_in vivo_, thus, a ratio of 1 with a patch containing fentanyl. The second group had a lower J_max_ (e.g., a patch of rivastigmine), and the third group containing nicotine as an active ingredient had a very high J_max_. The patches in the second group have penetration enhancers, which is the reason for the higher J_in vivo_. On the other hand, for the third group, the API is highly permeable, such that the J_max_ gets higher the in vitro study. Overall, the PAMPA model can be used to examine the permeability of the patches [93]. Balázs et al., compared the permeation profiles of the ibuprofen formulation in the PAMPA and human skin using vertical Franz cells. The correlation coefficients ranged from 0.88 to 0.99 for the PAMPA and Franz cells with human skin. Interestingly, the standard deviation in the permeation profiles from Franz cells was higher than the standard deviation observed in the PAMPA. The PAMPA model minimizes the variation in permeation due to inconsistent skin preparation, which is observed in Franz cells. Further, the authors claimed the superiority of the PAMPA over synthetic membranes while using the penetration enhancers like Transcutol^®^ and sucrose laurate D-1216. The synthetic membranes are hampered by sucrose laurate, whereas the permeation can still be accessed using the PAMPA. Moreover, the PAMPA could differentiate the depot phenomena of Transcutol^®^ from gel formulations, but a synthetic membrane fails to do so [94,95].

A comparative permeation study of niacinamide in Franz diffusion cells (with porcine or human skin) and the PAMPA model showed a linear correlation in the cumulative amount of niacinamide permeated with a finite dose in both the setups [96]. In a recent study by Kollmer et al., the compatibility of the skin-PAMPA membrane with lipophilic solvents/penetration-enhancers (isopropyl myristate, diisopropyl adipate, dimethyl isosorbide, and propylene glycol); topical emulsions containing different emulsifier systems (dimethyl sulfoxide or ethanol); and organic receptor media additives was investigated. The conventional PAMPA assay in which a donor is placed at the bottom was compared against the modified PAMPA assay, where the donor was in the top plate. Methylparaben, ethylparaben, and propylparaben were used as both model permeants/internal standards. With the modified assay setup, a trend for higher paraben permeation was observed. The results indicated that the organic solvent did not harm the membrane during the 4 h of the study. Dimethyl sulfoxide and ethanol below 50% were found to be compatible with the skin-PAMPA membrane. No signs of extraction of stearic acid, cholesterol, and certramides was observed with dimethyl sulfoxide and ethanol under standard assay conditions [97]. These studies indicate the potential of the skin-PAMPA model as a reliable screening tool for topical formulations. More studies involving the testing of semi-solid formulations and transdermal patches should be conducted to realize the complete potential of skin-PAMPA.

Recently, the phospholipid vesicles-based permeation assay (PVPA) has also been introduced as a promising alternate for skin models. Engesland et al., have demonstrated the suitability of the PVPA model to mimic the skin with a compromised barrier [98]. Similarly, a tight layer of liposomes is immobilized on a filter for liposome-based artificial skin membranes (LASMs). Then, the filters are attached to filter inserts, loaded with the drug solution, and placed in the acceptor compartment for the permeation study [99]. For the first time, Zhang et al., used the LASM in the Franz cells for the evaluation of the permeation of five different compounds. This study demonstrated the good correlation of the drug permeation between the novel LASM and porcine skin [100]. Based on the above-mentioned studies, skin-PAMPA is able to mimic the barrier properties of the human stratum corneum to some extent and could be considered as a quick and cost-effective research tool for predicting passive human skin permeability coefficients. However, it must be noted that it lacks the biological complexity of the skin, such as proteins, appendages, corneocytes, lipids, and the layered structure of the epidermis. These constraints limit its use to mostly as a potential prescreening tool for topical and transdermal formulations.

### 4.3. Strat-M™ and Other Artificial Membranes

**Figure 4:**
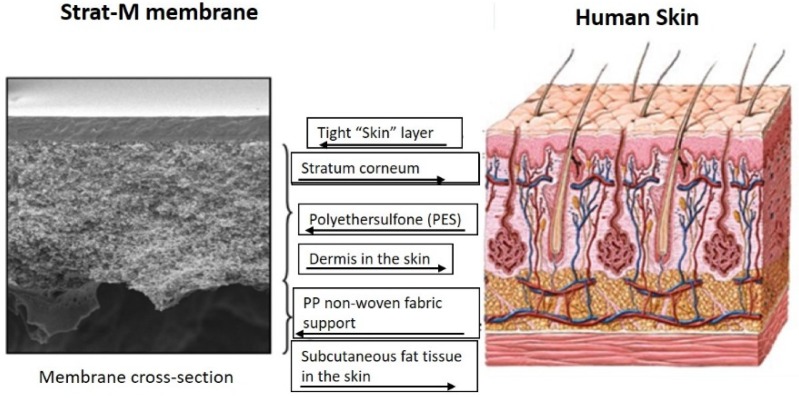
Comparison of Strat-M™ membrane and the human skin. Reproduced with permission from Haq et al. [102].

Synthetic artificial membranes are designed to mimic the human skin and offer a simple and reproducible alternative to human and animal skins. Unlike a biological skin, synthetic membranes can be easily procured and stored. They also reduce the variability in drug permeations that is associated with the utilization of biological skin [101]. These advantages, along with ethical concerns, have led scientists to look for synthetic membranes. The majority of studies involving synthetic membranes for transdermal delivery have reported the use of silicone-based membranes such as Silatos™, Silastic^®^, and polydimethylsiloxane [21]. Strat-M™ (Merck Millipore, Burlington, Massachusetts, USA), composed of multiple layers of polyester sulfone, is a recent addition to synthetic membranes. Like human skin, the Strat-M™ membrane has multiple layers with varied diffusivity, as shown in Figure 4. The outer layer consists of two layers of polyethersulfone (PES, more resistant to diffusion), while the bottom layer is a more diffusive polyolefin layer. The rate-limiting layer for permeation through the skin is the outermost epidermal layer, whereas, in artificial membranes, the rate-limiting layer is not explicit. It is challenging to separate the layers of the Strat-M™ membrane to study the drug entrapped in each layer.

**Figure 5:**
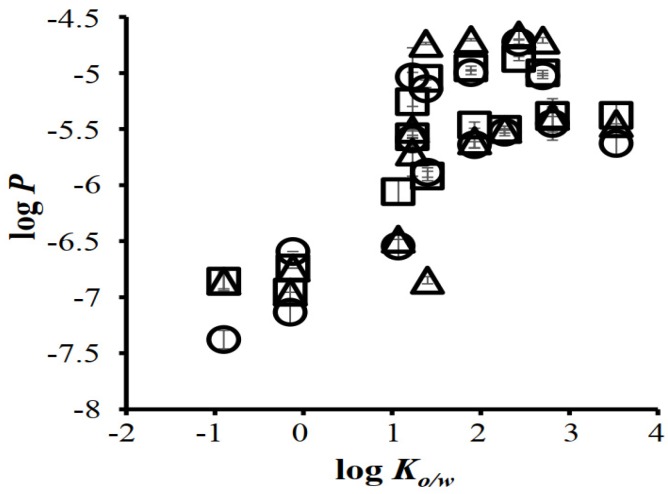
Relationships between the log *Ko/w* and log *P* of applied chemical compounds. Symbols: excised human skin (△), excised hairless rat skin (○), and Strat-M™ (□). All results are expressed as the mean ± S.E. (n = 3). *P*: permeability coefficient. Reproduced with permission from Uchida et al. [103].

**Figure 6:**
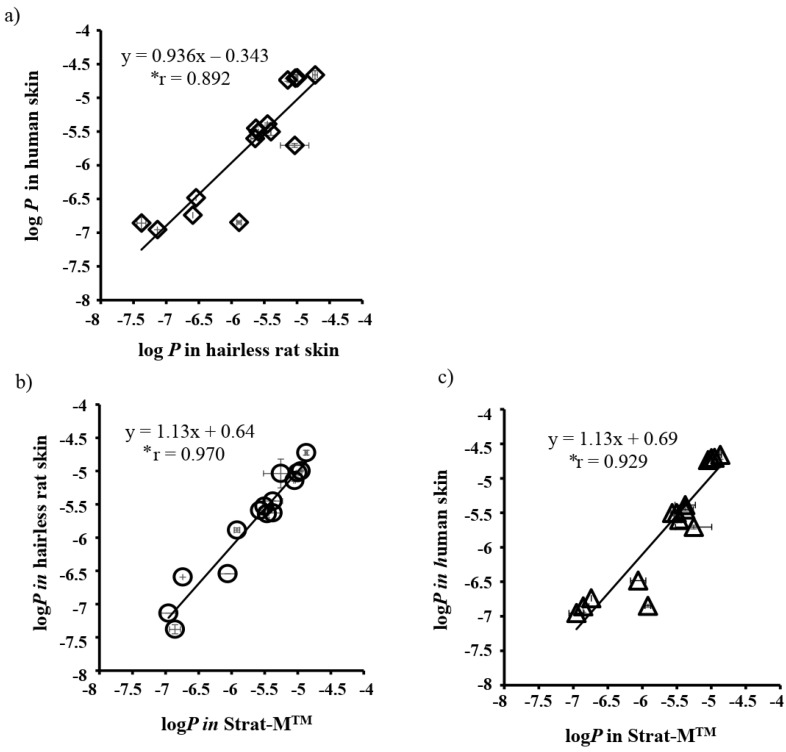
Relationships between log *P* values of human skin and rat skin or Strat-M™. (**a**) Human skin vs. Rat skin, (**b**) Rat skin vs. Strat-M™, (**c**) Human skin vs. Strat-M™. All results are expressed as the mean ± S.E. (n = 3). Pearson’s correlation coefficient showed significance (*p* < 0.05). Reproduced with permission from Uchida et al. [103].

Uchida et al., investigated the similarity of the Strat-M^TM^ membrane with human skin by comparing the permeability coefficients of ionized (*P_i_*) and unionized (*P_u_*) lidocaine between these two membranes. The *P_u_* was greater than *P_i_* in both the membranes. In the skin, *P_u_* was 177-fold higher than *P_i_*, whereas in the Strat-M™ membrane, it was only 43-fold higher. The tortuous aqueous-filled pores in the skin facilitate the permeation ionic drug molecules. Probably in the Start-M™ membrane, the hydrophilic pathway is less tortuous, which causes higher amounts of ionic drug permeation compared to the skin [103]. The same group also compared the permeation of 13 different chemical compounds (molecular weight (MW) between 152 and 289 Da and lipophilicity between −0.9 to 3.5) in the Strat-M™ membrane, human skin, and hairless rat skin. The permeability coefficients (*P*) of chemicals through the Strat-M™ membrane, human skin, and hairless rat skin membranes are shown in Table 1. The log *K_o/w_* of the compounds increased with increasing log *P* values in all three membranes. The log *P* values obtained in the Strat-M™ were close to those in human and rat skins (Figure 5). The relationship between the log *P* in human and rat skins, in rat skin and Strat-M™, and in human skin and Strat-M™ is shown in Figure 6a–c, respectively. A good relationship was observed between the human and rat skin data (Figure 6a) and between rat and human skin, which strongly indicates that an excised hairless rat could be used as an alternative to human skin. Though the Strat-M™ and human or rat skin also showed a good relationship, the slopes were slightly greater than the unity. This was attributed to the faster permeation of chemical compounds through the Strat-M™ compared with human and hairless rat skins [103].

In a later study, the effects of the physicochemical properties (solubility, viscosity, wettability, surface tension, and uptake) of 25 esters in the permeation of the four model compounds (caffeine, aminopyrine, benzoic acid, and flurbiprofen) across the Strat-M™ membrane and silicone membrane were reported. The permeation was higher for the model compounds when the esters with low molecular weights and surface tensions were used. Interestingly, for both the Strat-M™ and silicone membrane, the amounts of drugs permeated was not correlated to the solubility of the esters. Instead, there was a correlation with the other physiochemical properties of the esters, excluding caffeine. The caffeine permeation showed a correlation with the solubility of ester across the Strat-M™ membrane. Further, the permeation of the model compounds across the Strat-M™ and silicone membrane had a strong correlation [104]. The diffusion and partition parameters are linked to the permeation results of molecules across the membrane. The diffusion coefficient and partition coefficient are influenced not only by the nature of the molecules but also by the membrane used for the permeation. Therefore, one of the best ways to establish the relation of the artificial membrane with the skin is to compare the similarity of these parameters relative to the skin. The same group later used 15 model compounds (molecular weights between 122.12 and 270.80 Da and log* K_o/w_* between −1.50 to 3.86) dissolved in aqueous solutions to evaluate permeability parameters like the permeability coefficient (*P*), diffusion parameter (*DL^−2^*), and partition parameter (*KL*) in the silicone membrane, human skin, and hairless rat skin. The hydrophilic compounds like caffeine showed similar *P* values in all three membranes, whereas, in the silicone membrane, the amphiphilic compounds like aminopyrine and lipophilic compound-like flurbiprofen showed 10-and 100-fold higher *P* values, respectively. The log *P* value increased with the increasing lipophilicity in all membranes. Similarly, the log *KL* of all membranes increased with the increasing lipophilicity of the model compound. For the silicone membrane, there was a significant correlation of log *K_o/w_* with both log *P* and log *KL*. In contrast, the log *P* and log *K_o/w_* in the human and hairless rat skins could be represented by a sigmoid curve. There was a significant correlation between the log *KL* value in the silicone membrane and human skin (r = 0.815) and in the silicone membrane and hairless rat skin (r = 0.791). The *DL^−2^* values were 100-fold higher for the model compound in the silicone membrane compared to the human and hairless rat skins. Additionally, there was a significant correlation between the log *DL^−2^* values in the silicone membrane and human skin (r = 0.625) and between silicone membrane and the hairless rat skin (r = 0.699) [105].

Simon et al., demonstrated the superiority of the Strat-M™ membrane over other artificial membranes in an in vitro permeation study using an Exelon^®^ patch containing rivastigmine. Artificial membranes like silicone membranes (Sil2, Sil5, and Sil10); a polyethersulfone membrane (Strat-M™), and cellulose membranes (C_up_ and C_el_) were used in the study, and the results were compared with pig ear skin. The cumulative amount of rivastigmine permeated at the end of the study was not statistically different for artificial membranes and pigskins. The Sil10 membrane had a lesser amount of drug permeation until two hours, but later, the permeation was like the other membranes. This indicates that, despite the higher thickness of the Sil10 membrane, it does not cause higher diffusion resistance. The permeation profiles of the Strat-M™ membrane were statistically different from other membranes but were similar to the permeation profiles from the pig ear skin. The Strat-M™ membrane simulated the skin membrane better than the other artificial membranes. The Strat-M™ membrane had an in vitro-in vivo correlation of 0.991, whereas the other artificial membrane had 0.92 [106]. The Strat-M™ membrane possess synthetic lipids between the polymer layers, which have a porous structure with a thickness around 325 µm. The polymer layer on the Strat-M™ membrane has three layers based on the density. Thus, the Strat-M™ membrane provides the permeation gradient in terms of the pores and diffusivity, which enables it to mimic the biological skin membrane [103]. Recently, Kaur et al., used studied the permeation of amphotericin B across the Strat-M™ membrane and compared the results with human, pig, and rat skins. Nano-Formulations of amphotericin B were used in the study. The order of permeation of amphotericin B was rat skin > pig skin > Strat-M™ > human cadaver skin. The steady state flux from the nanoethogel, nanogel, drug solution, and marketed cream was evaluated across various biological membranes and the Strat-M™ membrane. Surprisingly, only the Strat-M™ membrane showed no statistically significant difference in the flux value with human cadaver skin, whereas the other biological membranes showed significant differences. Further, the morphological characteristic, such as thickness, pore size, surface morphology, and diameter of the Strat-M™, were found to be similar to the human skin based on scanning electron microscopy, transmission electron microscopy, and Brunauer-Emmett-Teller analyses [107]. A comparative study looking at the permeation of lidocaine from a nanostructured lipid carrier was conducted in a skin-PAMPA model and the Strat-M™. Both membranes correlated favorably with the heat-separated human epidermis in this study, with the Strat-M™ membrane sharing the most similarity in drug permeability profile to an ex vivo human skin model [108]. A summary of recent publications utilizing the Strat-M™ membrane in permeation studies is presented in Table 2.

Studies indicate that the Strat-M™ membrane is comparable to real human skin and can reasonably predict the permeation of both hydrophilic and lipophilic drugs. In general, hydrophilic compounds show higher permeations in the Strat-M™ membranes compared with lipophilic ones. Strat-M™ could be considered as a cheaper alternative for screening topical formulations and actives in the pharmaceutical and cosmetic industries. In most studies, the Strat-M™ membrane when compared with the human skin appeared to show a good correlation in the permeation data, suggesting its validity as a surrogate to human skin. Though synthetic membranes have a few advantages over human/animal skins, a clear-cut correlation to the human stratum corneum barrier function remains to be established, particularly in finite dose applications.

## 5. Conclusions and Future Directions

The human skin is regarded as a yardstick by the regulatory agencies in transdermal penetration and permeation studies. Despite ethical concerns, when human skin is not readily available, animal skin, (especially pig ear skin) is widely used as an alternative in percutaneous absorption studies. The animal skin models are also being used as important tools for screening a large number of chemicals, drug formulations, evaluations of skin permeation-enhancing mechanisms, and determining the rank of skin transports for a series of drug molecules. However, with the use of a large number of animal skin models in the literature, it becomes difficult to draw a valid comparison between the results obtained across various species. Academicians and pharmaceutical companies are focusing their efforts on developing standardized protocols and reliable alternatives to biological skins for conducting permeability studies. Artificial membranes to some extent replace human and animal skins because of their versatility in defining the thickness, composition, ease in handling and storage, inert nature, and reproducibility in the permeation data. On the negative side, the artificial membranes lack the superficial barrier that is observed in the stratum corneum and do not provide a correlation in the permeation data for human applications. Another challenge is the inability to recreate the heterogeneous nature of the skin, including cell metabolism and skin appendages. A holistic, collaborative approach between biologists, pharmacologists, and bioengineers is needed to further refine the existing models, which will ultimately lead to a reduction in animal experimentation and acceleration of drug research. Currently, the surrogate models discussed in this review could only be used as prescreening tools to rank drugs and formulations that could further be evaluated using a biological skin model. Skin surrogates could be relied on for identifying the permeability values and the percentages of the drugs absorbed. However, they fail to estimate the amount of drugs penetrated into different skin layers or the percentages of the doses absorbed. It is recommended that all the initial screening be done using skin surrogates, and the final products should be still tested using human skin for obtaining permeation data.

## Figures and Tables

**Table 1:** Permeability coefficients of chemicals through Strat-M™, hairless rat skin, and human skin. Values expressed as mean ± standard deviation (n = 3), Reproduced with permission from Uchida et al. [103].

Compounds	*P* (cm/s) × 10^−6^ Strat-M™	*P* (cm/s) × 10^−6^ Hairless Rat Skin	*P* (cm/s) × 10^−6^ Human Skin
MP	4.02 ± 0.6	2.39 ± 0.31	2.49 ± 0.03
EP	4.21 ± 0.2	2.96 ± 0.06	3.86 ± 0.49
PP	4.01 ± 0.16	3.63 ± 0.15	3.63 ± 0.15
BP	4.24 ± 0.34	2.31 ± 0.26	3.54 ± 0.18
ISMN	0.15 ± 0.02	0.07 ± 0.01	0.11 ± 0.01
AMP	0.87 ± 0.25	0.29 ± 0.11	0.33 ± 0.02
ISDN	3.14 ± 0.01	2.57 ± 0.15	3.14 ± 0.15
LCH (pH5.0)	0.14 ± 0.02	0.04 ± 0.01	0.05 ± 0.02
LCH (pH10)	5.55 ± 0.53	9.21 ± 3.68	1.97 ± 0.15
LCH (pH7.9)	1.2 ± 0.12	1.3 ± 0.11	0.14 ± 0.01
CAF	0.47 ± 0.07	0.26 ± 0.02	0.18 ± 0.02
M-PABA	8.95 ± 0.26	7.22 ± 0.17	18.4 ± 0.83
E-PABA	11.25 ± 0.41	10.6 ± 0.25	20.1 ± 2.24
P-PABA	13.55 ± 0.58	19.6 ± 1.61	22.3 ± 3.03
B-PABA	10.14 ± 0.53	9.49 ± 0.79	20.1 ± 3.02

Methyl paraben (MP), ethyl paraben (EP), n-propyl paraben (PP), n-butyl paraben (BP), isosorbide 5-mononitrate (ISMN), n-propyl p-aminobenzoate (P-PABA), n-butyl p-aminobenzoate (B-PABA), aminopyrine (AMP), Caffeine (CAF), lidocaine HCl (LCH), methyl p-aminobenzoate (M-PABA), ethyl p-aminobenzoate (E-PABA), isosorbide dinitrate (ISDN).

**Table 2:** Recent studies involving the use of synthetic membranes in permeation studies (partial listing).

Study	Important Findings	References
Topical delivery of a soluble form of naproxen from organogel.	Organogels containing a soluble form of naproxen could be a possible substitute to marketed topical products with the crystalline form of naproxen, as shown using a Strat-M™ membrane in the permeation study.	[109]
Topical delivery of a combination of glibenclamide and quercetin using chitosan nanogel.	Nanogel could be potential formulation for the delivery of an antidiabetic drug through the skin was demonstrated using a Strat-M™ membrane in the permeation study. Although such formulation enables controlled release of the drug, further improvement of the release mechanism is required for commercialization.	[110]
Super magnetic iron oxide nanoparticles dispersed in Pluronic F127 gel by topical delivery of nitric oxide (NO).	Permeation study carried using a Strat-M™ membrane demonstrated that the iron will not permeate from the nanoparticle dispersed in Pluronic.	[111]
Topical delivery of niclosamide (niclo) using octenylsuccinate hydroxypropyl phytoglycogen (OHPP) as a solubilizing agent.	Permeation study across the Strat-M™ membrane of niclo-OHPP solid dispersion was 5.3 times higher than niclosamide alone. This study can be used further in the development of the formulation of niclosamide.	[112]
Topical delivery of functional fragments of an AIMP1-derived peptide (AdP) in a hydrosol system.	A Strat-M™ membrane was used in the determination of in vitro deposition from FA-AdP hydrogel compared with FA-AdP alone. The in vitro deposition from optimized FA-AdP hydrosole in the Strat-M™ membrane was 127-fold higher than FA-Adp alone.	[113]
To develop a topical formulation of oxaprozin in liposomal or nanostructured lipid carriers (NLCs) formulation following a drug complexation with randomly-methylated-ß-CD and arginine.	The permeation of oxaprozin was screened first using the nitrocellulose membrane and later using the excised human skin. Both the formulations (deformable liposomes and NLCs) showed increased permeations compared to the plain drug. The deformable liposomes had significantly greater drug permeations than the NLCs across the human skin.	[114]
Topical delivery of benzocaine loaded with a polymeric nanoparticle/thermosensitive hydrogel system.	Benzocaine-loaded nanoparticles in hydrogel permeated through the artificial membrane Strat-M^™^, acting as a depot system for long duration action when applied on skin.	[115]
To check whether the use of lanolin in a synthetic membrane (Strat-M™ and nucleophore) would mimic the skin-barrier function using three different model drugs (lidocaine, diclofenac, and betamethasone).	The barrier function of the artificial membrane with lanolin was higher than without lanolin. The absorption of model drug substances on the lanolin-containing artificial membranes was found to be similar to that of the skin, which indicates the lanolin-containing membranes mimic topical active absorption.	[116]
To compare penetration profiles of lidocaine incorporated in the nanostructured lipid-carrier gel using different membranes (Strat-M™, skin-PAMPA, cellulose, and heat-separated human epidermis).	Drug permeability profile of lidocaine across the Strat-M™ membrane and skin-PAMPA correlated favorably with heat-separated human skin epidermis.	[108]
To evaluate the ability of the Strat-M™, isopropyl myristate, and certramides to predict the percutaneous absorption of six different compounds in saturated and unsaturated concentration in three different vehicles (water, ethanol, and propylene glycol).	The correlation of the absorption through membranes was drawn in relation to the porcine skin. The correlation was better with saturated concentrations than the unsaturated concentration of the compounds. Similarly, the Strat-M^™^ membrane had the best correlation, followed by certramides and IPM, for the amount remaining in the membrane and retained in the porcine skin.	[117]
To investigate the optimum condition of a capsaicin-loaded nano-emulsion formulation for topical application using the Strat-M™ membrane.	The nano-emulsion of sizes between 20 to 62 nm loaded with capsaicin successfully penetrated the layers in the Strat-M™ membrane. Thus, it was presumed to be suitable for evaluation in transdermal delivery.	[118]
To check whether the in vitro release profile of generic acyclovir creams is equivalent to the innovator product using different types of artificial membranes (Magna Nylon, Tuffryn membrane, Durapore, Nitrocellulose, Fluoropore, and Strat-M™).	The generic acyclovir cream formulations demonstrated similar release profiles, which were comparable to the reference product, Zovirax^®^. This study indicated the possibility of biowaivers for generic topical products based on in vitro studies using artificial membranes.	[119]
To examine the spreadability of the transdermal formulation in microneedle-treated and untreated skin by the study of parameters like the spreading radius, droplet height, and dynamic contact angle.	A Strat-M™ membrane was used as a control for the skin. The spreading parameters were lower for the lidocaine hydrogel compared to the lidocaine solution. The lidocaine hydrogel on the microneedle-treated skin resulted in slower dynamic reduction spreadability parameters because of microneedle cavities.	[120]
To determine the usefulness of synthetic membranes as an alternative of human and animal skins to evaluate the skin permeability.	Thirteen different compounds with molecular weights in the range of 152–289 and lipophilicity (log *K_o/w_*) in 0.9–3.5 were used in the permeation study through the Strat-M™, excised human skin, and hairless rat skin. The Strat-M™ had similar characteristics to that of the skin membrane in terms of the log *P* values, diffusion, and partition coefficient.	[103]
To develop an in vitro drug permeation test utilizing a synthetic membrane that can be a substitute for the permeation study where the animal or human skin is used.	Out of six different synthetic membranes used in the study, the Strat-M^®^ membrane adequately mimicked the barrier property of the skin, where the rivastigmine permeation profile through the Strat-M™ was similar to that of pig skin (R^2^ − 0.920). Additionally, the in vitro-in vivo correlation was linear (R^2^ − 0.991) for the Strat-M™ membrane.	[106]
To demonstrate the feasibility of incorporating the thermo-responsive nanogels in the semisolid gel or hydrogel film formulation to enable the adjustment in drug transport kinetics using diclofenac in a formulation containing gellan gum.	A Strat-M™ membrane was used as the substitute to the skin barrier in this study, which yielded high reproducible results with the ease of use. It was demonstrated that the combination of thermo-responsive nanogel with a semisolid gel or a hydrogel of diclofenac would result in a formulation that can provide fast drug penetration for rapid pain relief, as well as sustained drug delivery over a period.	[121]
To investigate the effect of 25 different esters in the permeation of four model compounds (caffeine, aminopyrine, benzoic acid, and flurbiprofen) with different polarity through the synthetic membranes (silicone and Strat-M™).	The amount of the model compounds that permeated through the silicon and the Strat-M™ membrane had significant correlation with the wettability, surface tension, and uptake of esters into the membrane. Therefore, the type of ester used as a vehicle is pivotal to control the skin permeability of topical formulations.	[104]
To formulate and evaluate the percutaneous absorption of a liquid crystal emulsion of retinyl palmitate through the skin barrier using a Strat-M™ membrane.	The liquid crystal emulsion showed increased retention at the membrane and high permeation to the acceptor chamber in a permeation study compared to the plain oil-water emulsion.	[122]
The aim of the study was to develop an ultra-deformable liposomal and microemulsion formulation for the transdermal delivery of clonazepam using cyclodextrin as a penetration enhancer.	A nitrocellulose membrane was used for preliminary screening of the formulation. Later, the best formulations were screened using animal skins. The permeability of microemulsion with cyclodextrin had a 4-fold higher permeability than of clonazepam. Similarly, the liposomes without cyclodextrin had a 2-fold higher permeability.	[123]
To develop a topical formulation of oxaprozin in liposomal or nanostructured lipid carriers (NLCs) formulation following a drug complexation with randomly-methylated-ß-CD and arginine.	The permeation of oxaprozin was screened first using the nitrocellulose membrane and later using the excised human skin. Both the formulations (deformable liposomes and NLCs) showed increased permeations compared to the plain drug. The deformable liposomes had significantly greater drug permeations than the NLCs across the human skin.	
